# Cathelicidin-Related Antimicrobial Peptide Negatively Regulates Bacterial Endotoxin-Induced Glial Activation

**DOI:** 10.3390/cells11233886

**Published:** 2022-12-01

**Authors:** Anup Bhusal, Youngpyo Nam, Donggun Seo, Won-Ha Lee, Kyoungho Suk

**Affiliations:** 1 Department of Pharmacology, School of Medicine, Kyungpook National University, Daegu 41944, Republic of Korea; 2 BK21 Plus KNU Biomedical Convergence Program, Department of Biomedical Science, School of Medicine, Kyungpook National University, Daegu 41944, Republic of Korea; 3 BK21 FOUR KNU Creative BioResearch Group, School of Life Sciences, Kyungpook National University, Daegu 41566, Republic of Korea; 4 Brain Science and Engineering Institute, Kyungpook National University, Daegu 41944, Republic of Korea

**Keywords:** CRAMP, LPS, microglia, astrocyte, neuroinflammation

## Abstract

Recent studies have suggested that mouse cathelicidin-related antimicrobial peptide (CRAMP) and its human homologue leucine leucine-37 (LL-37) play critical roles in innate immune responses. Here, we studied the role of mouse CRAMP in bacterial endotoxin lipopolysaccharide (LPS)-induced neuroinflammation. CRAMP peptide treatment significantly inhibited LPS-mediated inflammatory activation of glial cells in culture. In the animal model of LPS-induced neuroinflammation, CRAMP expression was highly induced in multiple cell types, such as astrocytes, microglia, and neurons. Injection of exogenous CRAMP peptide significantly inhibited inflammatory cytokine expression and the reactivity of glial cells in the mouse brain following intraperitoneal or intracerebroventricular LPS administration. Altogether, results of the study suggest that CRAMP plays an important part in containment of LPS-induced neuroinflammatory responses, and that CRAMP can be exploited for the development of targeted therapies for neuroinflammatory conditions associated with bacterial infection.

## 1. Introduction

Cathelicidins are a diverse group of antimicrobial peptides essential for the defense response against infection and tissue injury [1]. Cathelicidin-related antimicrobial peptide (CRAMP) and its human ortholog, leucine leucine-37 (LL-37), play active roles in diverse immune and inflammatory responses to microbial infection. Specifically, the microbicidal activity of CRAMP is primarily mediated by the disruption of the integrity of microbial membranes [2]. Additionally, CRAMP exerts its antimicrobial action by interacting with several components of microorganisms [3,4].

Lipopolysaccharide (LPS), an endotoxin, is a powerful mediator of systemic inflammation. Various studies have shown that CRAMP is expressed in response to different infections where it binds to LPS and neutralizes its biological action [5,6,7]. Furthermore, CRAMP affects inflammatory response by acting on the Toll-like receptors (TLR) pathway or by binding to intracellular glyceraldehyde-3-phosphate dehydrogenase (GAPDH) [8,9]. However, CRAMP previously potentiated neuroinflammation in a mouse model of multiple sclerosis; these findings are supported by other reports [10,11]. Given this context, it is important to determine whether CRAMP exhibits pro- or anti-inflammatory activity in the central nervous system (CNS) in relation to LPS response.

Here, we used in vitro and in vivo models to investigate the role of CRAMP on LPS-induced neuroinflammation. Our study revealed that CRAMP suppressed the inflammatory activation of cultured glial cells following LPS treatment. The findings were recapitulated in animal model of LPS-induced neuroinflammation. Our study suggests that the CRAMP peptide possesses an ability to suppress the inflammatory activation of glia in the brain following bacterial infection. Our results also imply that CRAMP has a multifaceted role in brain inflammation.

## 2. Materials and Methods

### 2.1. Animals

Samtako Bio Korea provided the C57BL/6 mice used in the study. All animal tests were carried out with male mice aged 8 weeks. Mice were kept in standard light/dark cycles (12 h/12 h) and all experiments were conducted as per protocols approved by Institutional Animal Care and Use Committee (KNU 2019-09).

### 2.2. Animal Model of Neuroinflammation

Adult male (8 weeks) C57BL/6 mice were injected with LPS via two different routes: intraperitoneal (i.p.) or intracerebroventricular (i.c.v) for neuroinflammation model. For the systemic injection method, mice were administered with LPS (5 mg/kg) via the i.p. route [12]. An identical volume of saline was delivered in the control animals. The mice were anesthesized and then sacrificed at 24 h after LPS injection. For the direct brain (i.c.v.) injection method, LPS (5 µg/mouse) was delivered (0.2 µL/min) into the lateral ventricle (anteroposterior, −0.02 mm; mediolateral, 1.0 mm; dorsoventral, −2.0 mm) using Hamilton syringe. The CRAMP peptide was delivered via the same route (i.c.v.). The CRAMP- and/or LPS-injected sides of the mouse brains were used for biological assays.

### 2.3. Glial Cell Culture

Glial cell was cultured as per the protocols published before [10]. The BV-2 microglia cells were grown at 37 °C in Dulbecco’s modified Eagle medium with 5% heat-inactivated fetal bovine serum (FBS), 100 U/mL penicillin, and 100 g/mL streptomycin (Gibco, Grand Island, NY, USA). Three-day-old mouse brains were mechanically shattered and homogenized for primary microglial cell cultures. The mixed glial cells were sown in culture flasks and cultured in DMEM supplemented with 10% FBS in an incubator at 37 °C with 5% CO_2_. Primary microglia were obtained from mixed glial cells after 14 days of culture using a gentle trypsinization technique, and they were kept alive in DMEM supplemented with 10% FBS and penicillin-streptomycin [13]. For astrocyte culture, the mixed glial culture was mechanically agitated overnight at 200 rpm. The culture medium was discarded and astrocytes were dissociated using trypsin-ethylenediamine tetraacetic acid (Invitrogen, Eugene, OR, USA) before being collected by centrifugation at 1200× *g* rpm for 10 min. The acquired primary astrocytes were cultured in 10% FBS, high-glucose DMEM, and penicillin-streptomycin medium.

### 2.4. Immunofluorescence Staining

The brain tissues obtained from the experimental animals were fixed using 4% paraformaldehyde. The fixed tissues were washed with phosphate-buffered saline (PBS) and put in sucrose solution and embedded in an optimal cutting temperature compound (Tissue-Tek; Sakura Finetek, Torrance, CA, USA). Tissue sections (20 µm thick) were prepared and rinsed in PBS followed by incubation with the following antibodies: goat Iba-1 (Iba-1; 1:500; Novus Biologicals, Littleton, CO, USA), mouse anti-GFAP cocktail (1:500; BD Biosciences, San Jose, CA, USA), mouse anti-neuronal nuclei (NeuN; 1:500, EMD Millipore Corp., Temecula, CA, USA) and rabbit anti-CRAMP (1:200; Novus Biologicals, Littleton, CO, USA). Following overnight incubation, the brain sections were washed and treated with fluorescein isothiocyanate (FITC)-conjugated or Cy3-conjugated secondary antibodies (1:200; Jackson ImmunoResearch, West Grove, PA, USA) for 2 h. Next, tissue sections were washed and mounted with medium containing 4′,6-diamidino-2-phenylindole (DAPI, Vector Laboratories, Burlingame, CA, USA). Images of the tissue sections were taken under a fluorescence microscope (Leica Microsystems, DM2500, Wetzlar, Germany). DAPI was visualized using excitation at 360 nm and emission at 470 nm, FITC with excitation at 490 nm and emission at 525 nm, and Cy3 with excitation at 557 nm and emission at 576 nm.

### 2.5. Quantification of Immunostaining

The images from three coronal sections of each mouse brain (n = 3; between −1.70 mm and −2.18 mm from bregma) [14] were chosen for quantification. GFAP-, Iba-1-, or NeuN-positive cells colocalized with CRAMP in the dorsal CA1 region of the hippocampus were counted manually and expressed as the cell number per mm^2^ area. Images were acquired under a 20× objective (coordinates shown in Figure S1A). Similarly, for the measurement of immunoreactivity, images of brain hippocampal sections acquired under 5× objective (coordinates shown in Figure S1B) were imported into ImageJ (NIH, Bethesda, MD, USA), and relative intensity measured and expressed as fold change.

### 2.6. Measurement of Nitric Oxide Production

The nitrite levels were assessed in the culture media using Griess assay to estimate nitric oxide (NO) production. Cultured cells were stimulated with LPS (100 ng/mL) in the presence of the CRAMP peptide. Following incubation, cell culture medium was mixed with an equal volume of Griess reagent in a 96-well plate, and light absorbance at 540 nm was measured.

### 2.7. Assessment of Cell Viability

As previously described [9], cell viability was determined using the 3-[4,5-dimethylthiazol-2-yl]-2,5 diphenyl tetrazolium bromide (MTT) assay. After the indicated period of LPS or CRAMP pep-tide treatment, the culture medium was discarded and MTT (0.5 mg/mL in 0.1M PBS) was added to the cells for 2 h at 37 °C. The resulting formazan crystals were dissolved in dimethyl sulfoxide, and the absorbance at 570 nm was measured.

### 2.8. Enzyme-Linked Immunosorbent Assay (ELISA)

Tumor necrosis factor-alpha (TNF-α) and C-X-C motif chemokine ligand 10 (CXCL10) levels in culture media were measured using mouse TNF-α and CXCL10 ELISA kits (R&D Systems, Minneapolis, MN, USA), as described previously [15]. The assays were performed according to the manufacturer’s instructions. All measurements were performed in duplicate.

### 2.9. Quantitative Real-Time Polymerase Chain Reaction

Qiazol lysis reagent (Qiagen, Germantown, MD, USA) was used to extract RNA from cell culture and tissue samples. Total RNA (2 µg) from each sample was reverse-transcribed into cDNA using the First Strand cDNA Synthesis Kit (MBI Fermentas, Hanover, Germany). Real-time reverse transcription-polymerase chain reaction (RT-PCR) was conducted using a One-step PrimeScript^TM^ RT-PCR Kit (Perfect Real-Time; Takara Bio Inc., Tokyo, Japan) and the ABI Prism 7000 sequence detection system (Applied Biosystems, Foster City, CA, USA), according to the manufacturer’s instructions. The 2^−ΔΔCt^ method was used to calculate the relative changes in gene expression [16], and *Gapdh* was used as an internal control. The nucleotide sequences of the primers used for the mouse samples in the real-time RT-PCR were as follows: *Cramp:* forward 5′-AAT TTT CTT GAA CCG AAA GGG-3′, reverse 5′-TGT TTT CTC AGA TCC TTG GGA GC-3′; *Tnf*: forward 5′-ATG GCC TCC TCA TCA GTT C-3′, reverse 5′-TTG GTT TGC TAC GAC GTG-3′; *Il1b*: forward 5′-AAG TTG ACG GAC CCC AAA AGA T-3′, reverse 5′-TGT TGA TGT GCT GCT GCG A-3′; *Il6*: forward 5′-AGT TGC CTT CTT GGG ACT GA-3′, reverse 5′-TCC ACG ATT TCC CAG AGA AC-3′; *Cxcl10:* forward 5′-AAG TGC TGC CGT CAT TTT CT-3′, reverse 5′-GTG GCA ATG ATC TCA ACA CG-3′; *Gapdh*: forward 5′-TGG GCT ACA CTG AGC ACC AG-3′, reverse 5′-GGG TGT CGC TGT TGA AGT CA-3′.

### 2.10. Statistical Analysis

All statistical analyses were performed using GraphPad Prism software (version 8, La Jolla, CA, USA). Results were presented as mean ± SEM, and Student’s *t*-test or one/two-way ANOVA with Tukey’s post hoc test was used for statistical comparison. In the graphs of in vivo experiments, each data point represented an individual animal. To confirm results, the in vitro experiments were repeated at least twice. *p* < 0.05 was deemed statistically significant.

## 3. Results

### 3.1. CRAMP Peptide Treatment Attenuates LPS-Induced Production of Inflammatory Mediators in Cultured Glial Cells

**Figure 1:**
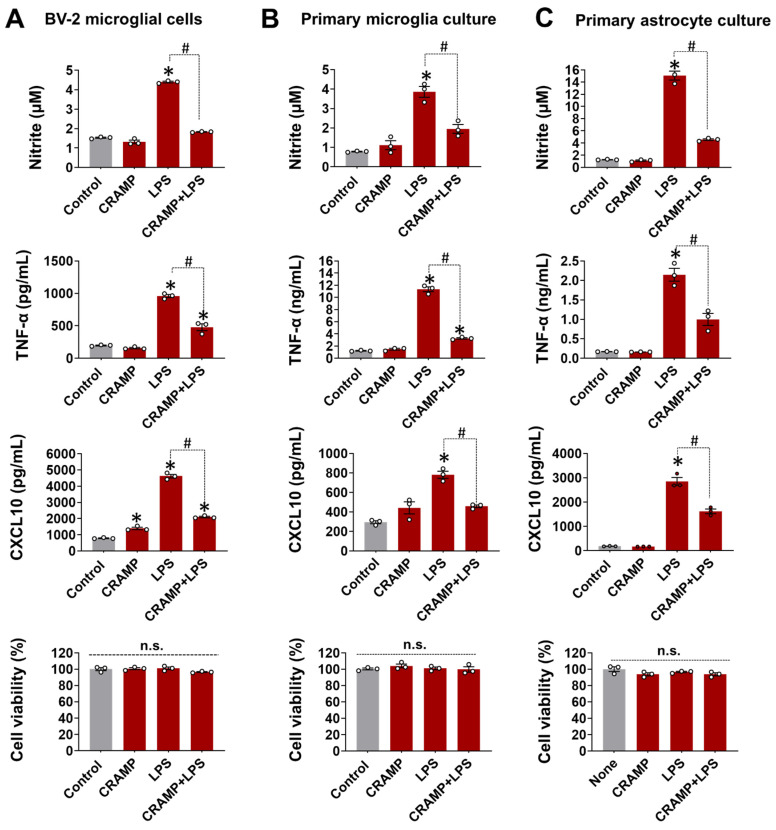
CRAMP peptide treatment suppresses LPS-induced glial activation. The effect of CRAMP peptide (30 μg/mL) treatment on LPS-induced production of NO, TNF-α, and CXCL10 was determined in BV-2 cells (**A**), primary microglia (**B**), and astrocytes (**C**) at 24 h, either by nitrite measurement or ELISA. MTT assay was utilized to assess cell viability, which showed no difference between treatment groups. Data are mean ± SEM; n = 3; * *p* < 0.05 versus control; # *p* < 0.05 between specified groups. One-way ANOVA with Tukey’s post hoc test. n.s., not significant.

To check CRAMP effect on LPS-stimulated glial cells, the production of inflammatory mediators was measured following treatment with LPS and CRAMP. CRAMP peptide treatment significantly inhibited the production of NO, TNF-α, and CXCL10 in BV-2 microglial cells (Figure 1A), primary microglia (Figure 1B), and astrocyte cultures (Figure 1C). These data indicate that CRAMP suppresses LPS-mediated inflammatory activation of glial cells in culture.

**Figure 2:**
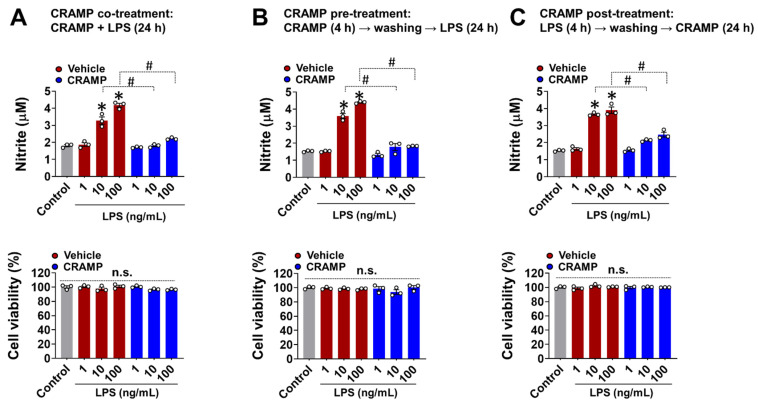
Effect of different CRAMP peptide treatment conditions on NO production in LPS-treated BV-2 microglial cells. The Griess assay show that CRAMP peptide treatment (30 μg/mL) attenuates LPS-induced NO production in BV-2 microglial in different experimental settings: (**A**), co-treatment; (**B**), pre-treatment; (**C**), post-treatment. MTT assay was utilized to assess cell viability, which showed no significant difference between treatment groups. Data are mean ± SEM; n = 3; * *p* < 0.05 versus control; # *p* < 0.05 between the specified groups. One-way ANOVA with Tukey’s post hoc test. n.s., not significant.

Studies have shown that CRAMP-mediated inflammatory responses generally depend on the timing and sequence of exposure to CRAMP and other stimuli [17]. To address this aspect, we performed CRAMP peptide co-, pre-, and post-treatment to LPS-stimulated BV-2 microglial cells and measured NO levels as a representative molecule for glial activation [10,12,18]. In all of these experimental settings, the CRAMP peptide significantly inhibited the LPS-induced production of NO in BV-2 microglial cells (Figure 2A–C). We also found that the CRAMP peptide post-treatment inhibited LPS-induced TNF-α production in cultured primary microglia and astrocytes, similar to that of the co-treatment condition (Figure S2). Our data revealed that CRAMP-mediated inhibition of glial LPS response does not depend on the direct physical interactions between CRAMP and LPS. These data further showed that the sustained presence of CRAMP was not required for the suppression of LPS response in glial cells.

### 3.2. Cramp mRNA and CRAMP Protein Are Expressed in the Brain of LPS-Injected Mice

**Figure 3:**
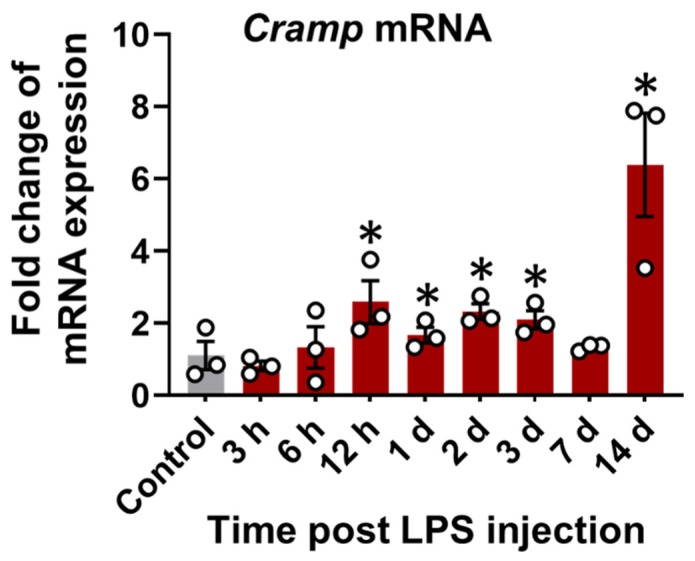
*Cramp* mRNA expression in LPS (i.p.)-injected mice. The *Cramp* mRNA level was determined in the brain of LPS (i.p.)-injected mice at different time points. Data are mean ± SEM; n = 3; * *p* < 0.05 versus control. One-way ANOVA with Tukey’s post hoc test. n.s., not significant.

*Cramp* mRNA expression was first evaluated in the brain tissue of LPS (i.p.)-injected mice. A time-dependent increase in *Cramp* mRNA expression was observed in the brains of LPS (i.p.)-injected mice relative to control group (Figure 3).

**Figure 4:**
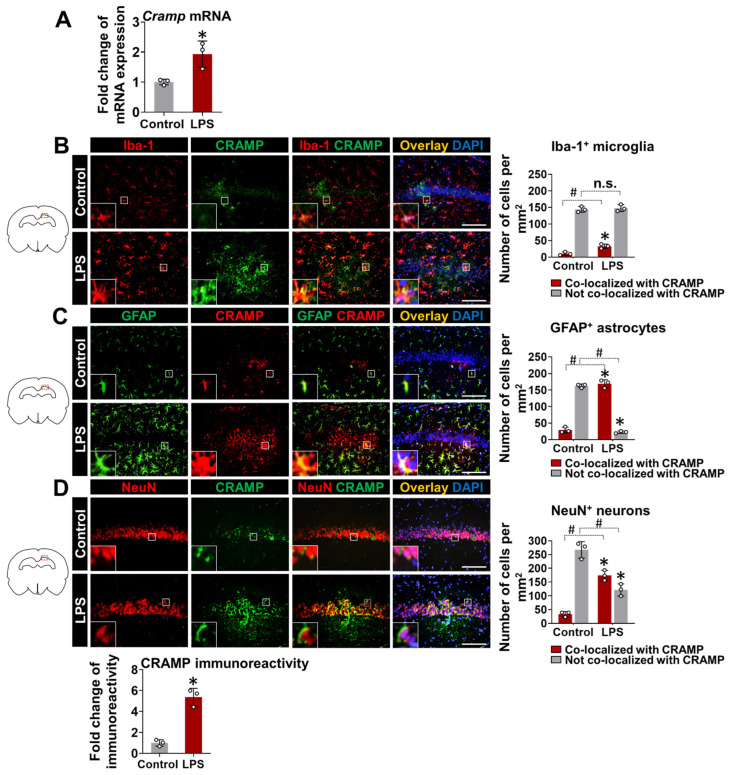
*Cramp* mRNA and CRAMP protein expression in LPS (i.c.v.)-injected mice. (**A**) The *Cramp* mRNA level was determined in the brain of LPS (i.c.v.)-injected mice at 24 h. (**B**–**D**) Immunofluorescence analysis of mouse hippocampus sections to check CRAMP expression in microglia (**B**), astrocytes (**C**), and neurons (**D**). Scale bars indicate 200 μm. Quantification of colocalization between CRAMP expression and each cell type is shown in graphs (*right*). The fold change in CRAMP protein immunoreactivity was also measured following LPS (i.c.v.) injection (*bottom*). Data are mean ± SEM; n = 3; * *p* < 0.05 versus control; # *p* < 0.05 between the specified groups. Student’s *t*-test (**A**) and Two-way ANOVA (**B**–**D**) with Tukey’s post hoc test. i.c.v., intracerebroventricular. n.s., not significant.

To further confirm our findings and check the direct effect of LPS on CRAMP expression, we delivered LPS through the i.c.v. route in mice. LPS delivery into animal brains significantly upregulated *Cramp* mRNA expression at 24 h (Figure 4A). We also performed immunofluorescence staining of brain sections from LPS (i.c.v.)-injected mice. CRAMP protein expression colocalized with Iba-1^+^ microglial cells (Figure 4B), GFAP^+^ astrocytes (Figure 4C), and NeuN^+^ neurons (Figure 4D). Our quantitative analysis of CRAMP protein immunoreactivity and its localization in different cell types showed that CRAMP protein was highly expressed in astrocytes and neurons, compared to microglia cells, in LPS (i.c.v.)-injected mouse brains (Figure S3), which was similar to our previous findings [10]. Immunoreactivity for CRAMP protein was limited in the brain sections of control animals (Figure 4B–D).

### 3.3. CRAMP Peptide Administration Attenuates Neuroinflammation in LPS-Injected Mice

**Figure 5:**
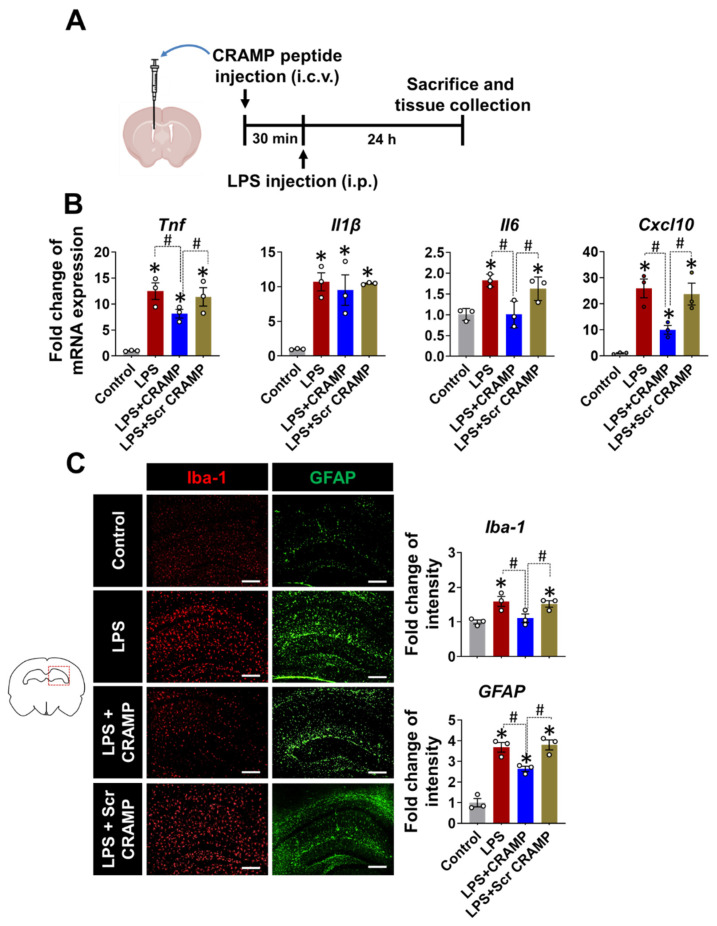
CRAMP peptide administration attenuates LPS (i.p.)-induced neuroinflammation. (**A**) Scheme of the experimental timeline with the routes of CRAMP peptide and LPS administrations. (**B**) The mRNA levels of inflammatory mediators in the brains of LPS-injected (i.p.) mice following administration of CRAMP peptide (i.c.v.) or control peptide of scrambled sequence (Scr CRAMP). (**C**) The hippocampal region of the brain sections was stained with GFAP or Iba-1 antibodies. Scale bars indicate 400 μm. The adjacent graphs represent the quantitative analysis of immunoreactivity of glial cells. Data are mean ± SEM; n = 3; * *p* < 0.05 versus control animals; # *p* < 0.05 between the specified groups. One-way ANOVA with Tukey’s post hoc test. i.p., intraperitoneal; i.c.v., intracerebroventricular.

Based on the increased expression of *Cramp* mRNA and CRAMP protein in the brains of LPS-injected mice, we next evaluated the effect of CRAMP peptide on neuroinflammation. We infused the CRAMP peptide through the i.c.v. route into LPS (i.p.)-injected mice (Figure 5A). The i.c.v. delivery of CRAMP peptide attenuated LPS (i.p.)-induced neuroinflammation, as shown by a decrease in the LPS-induced expression of inflammatory cytokines, such as *Tnf*, *Il6*, and *Cxcl10* mRNA (Figure 5B). However, we did not observe significant CRAMP effect on LPS-induced *Il-1β* mRNA levels. Furthermore, immunofluorescence analysis showed a decrease in LPS-induced immunoreactivity of Iba-1 and GFAP (Figure 5C) in the brains of CRAMP peptide-injected mice, but not in scrambled peptide-injected animals. These results suggest that CRAMP attenuates neuroinflammation in LPS (i.p.)-injected mice.

**Figure 6:**
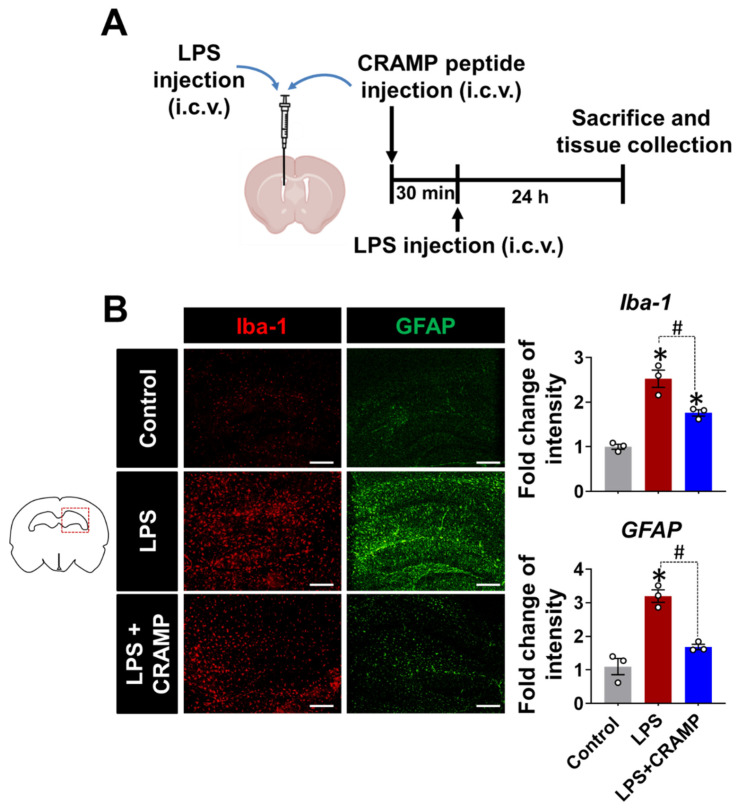
CRAMP peptide administration suppresses LPS (i.c.v.)-induced gliosis in mouse brain. (**A**) Scheme of the experimental timeline with the routes of CRAMP peptide and LPS administrations. (**B**) The hippocampal region of the mouse brain sections was stained with GFAP or Iba-1 antibodies. Scale bars indicate 400 μm. The adjacent graphs represent the quantitative analysis of the immunoreactivity of glial cells. Data are mean ± SEM; n = 3; * *p* < 0.05 versus control animals; # *p* < 0.05 between the specified groups. One-way ANOVA with Tukey’s post hoc test. i.c.v., intracerebroventricular.

Similar anti-inflammatory effects of CRAMP peptide were observed in the LPS (i.c.v.)-injected mice (Figure 6). We found that the LPS injection (i.c.v.)-induced increase in Iba-1 and GFAP immunoreactivity was reduced by CRAMP peptide administration (Figure 6B). Collectively, our data showed that CRAMP inhibited LPS-induced neuroinflammation in vivo.

## 4. Discussion

This study demonstrated an inhibitory effect of CRAMP on LPS-induced neuroinflammation. CRAMP treatment diminished the LPS effect in cultured glial cells. These findings were recapitulated in the in vivo study, where CRAMP peptide administration suppressed LPS-induced neuroinflammation in mice. These findings suggest that CRAMP expression in the brain may be a part of the CNS innate immune response to bacterial infection, which may prevent excessive neuroinflammation.

CRAMP plays an immunomodulatory role in various infections and neuroinflammatory conditions. Here, we show that CRAMP treatment suppressed the LPS effect in glial cells activation. Our findings are in line with previous reports showing the LPS-neutralizing capacity of the CRAMP/LL-37 peptide [6]. However, the CRAMP role in inflammation has been context-dependent, as the effects of CRAMP on TLR4 responses might depend on the experimental conditions of CRAMP and LPS exposure. The pretreatment of macrophages and monocytes with LPS has been shown to potentiate inflammasome activation and the production of TNF-α, interleukin-1β, and interleukin-18 following CRAMP exposure [19,20,21]. We also performed similar pre- and post-treatments with CRAMP peptide in LPS-stimulated microglial cells. We did not observe any differences between pre-, post-, and cotreatment conditions; in all conditions tested, CRAMP diminished the production of inflammatory mediators induced by LPS. Thus, the ability of CRAMP to suppress LPS response in glia was not dependent on the sequence of CRAMP and LPS exposure.

LPS injection in mice induced marked expression and upregulation of CRAMP in both glial and neuronal cells in the brain. However, CRAMP expression was also observed in control mice. These results led us to propose the possibility of a CRAMP standby expression and turnover, which can provide an immediate response to immune challenges, such as bacterial infection. Exogenous CRAMP peptide administration inhibited the LPS-induced inflammatory response in mouse brains following LPS i.p. and i.c.v. injection. In particular, in the former model, a similar CRAMP-mediated suppression of the LPS response was observed, even in the absence of a direct interaction between CRAMP and LPS.

Cathelicidins are key antimicrobial effector proteins that protect body surfaces from invasive bacterial infections as part of the innate immune system [22]. It has been previously shown that CRAMP can be locally generated at the sites of inflammation. Our findings extend the expression and role of CRAMP in the CNS as a modulator of the innate immune response to incoming bacterial insult. Our findings illuminate the role of CRAMP in the CNS during distinct infective states. However, further studies are required to fully comprehend its involvement in neuroinflammation and diverse CNS conditions.

## Data Availability

The data supporting the findings of this study are available from the corresponding author upon reasonable request.

## Supplementary Materials

The following supporting information can be downloaded at: https://www.mdpi.com/article/10.3390/cells11233886/s1. Figure S1. Schematic representation of brain sections used for immunostaining. Figure S2. The effect of CRAMP peptide post-treatment on the production of inflammatory mediators in LPS-stimulated glial cells. Figure S3. Cellular contribution of CRAMP expression in the hippocampus of LPS-injected mice.
