# Supplementary material for: Cathelicidin-Related Antimicrobial Peptide Negatively Regulates Bacterial Endotoxin-Induced Glial Activation
Source: Cells. 2022 Dec 1;11(23):3886. doi: 10.3390/cells11233886 (PMC9738883; doi:10.3390/cells11233886)
Supplement: Supplementary file 1 [file cells-11-03886-s001.zip › cells-1961980-SM.pdf]

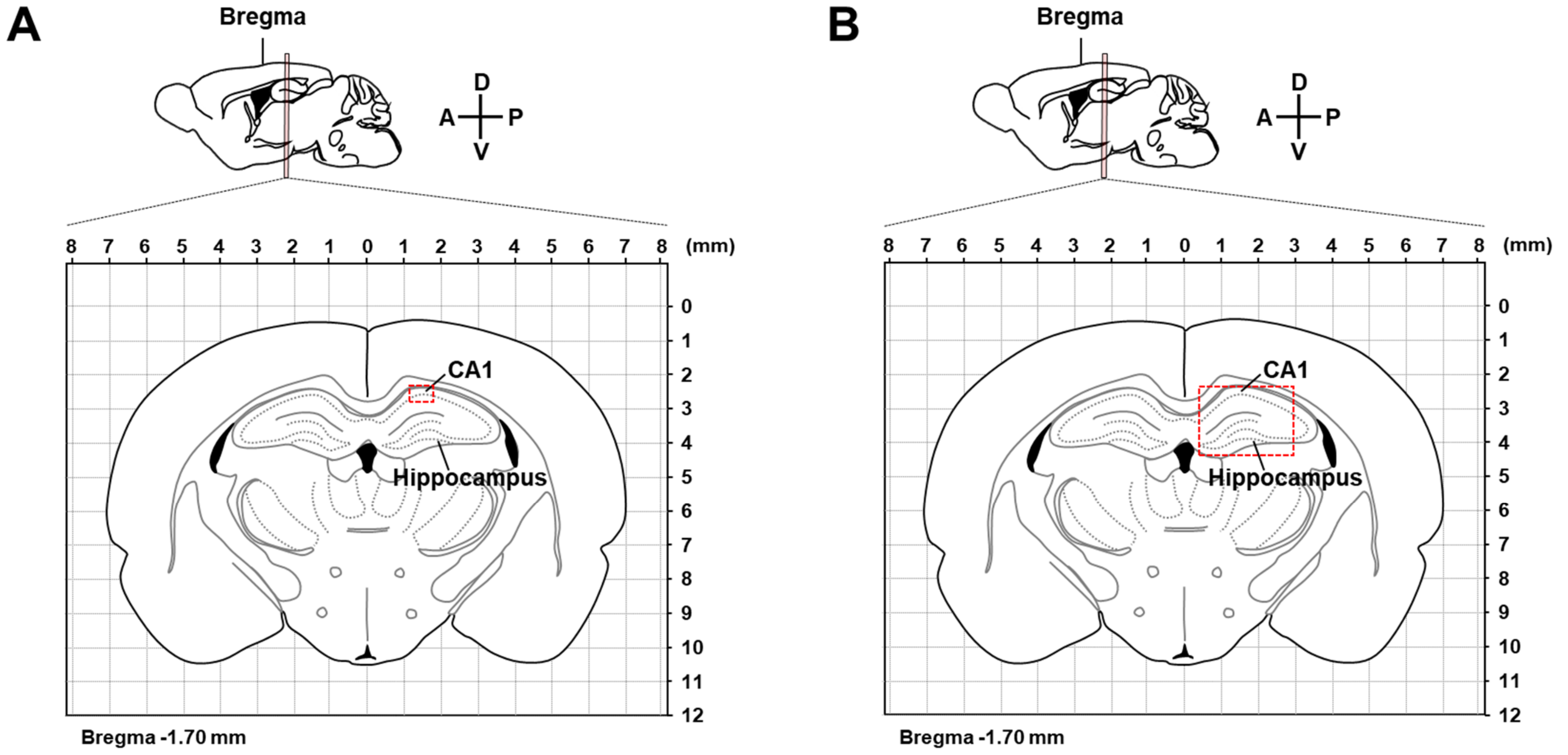

Figure S1. Schematic representation of brain sections used for immunostaining. (**A**) The coordinates of the hippocampal region of mice brain imaged under 20× objective for the study of CRAMP localization in different cell types. (**B**) The coordinates of the hippocampal region imaged under 5× objective to study the reactive gliosis in LPS-injected mice.

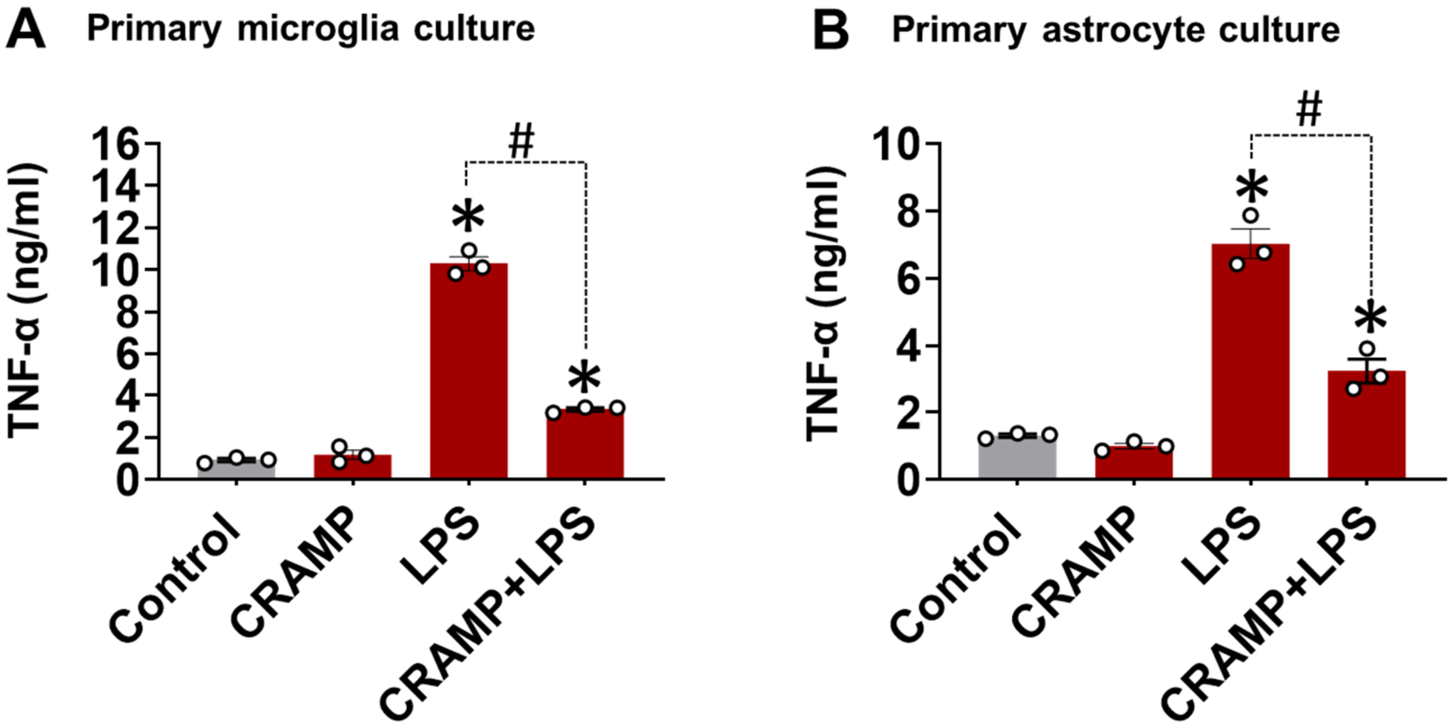

Figure S2. The effect of CRAMP peptide post-treatment on LPS-stimulated glial cells. The effect of CRAMP peptide (30 μg/mL) post-treatment on LPS-induced production of TNF-α was determined in primary microglia (**A**) and astrocyte culture (**B**). Data are mean ± SEM; n = 3; * $p < 0.05$ versus control; # $p < 0.05$ between the specified groups. One-way ANOVA with Tukey's post hoc test.

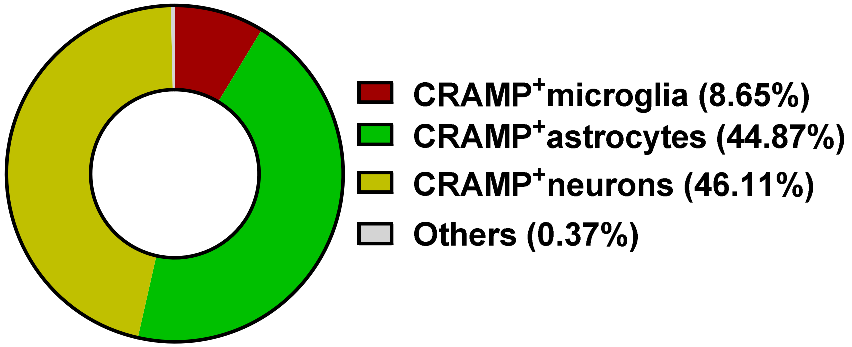

Figure S3. Cellular contribution of CRAMP expression in the hippocampus of LPS-injected mice.
